# Tri-State Medical Society of Alabama, Georgia, and Tennessee

**Published:** 1894-10

**Authors:** 


### Society Reports.

### TRI-STATE MEDICAL SOCIETY OF ALABAMA, GEORGIA, AND TENNESSEE.—SIXTH ANNUAL SESSION.

### FIRST DAY, TUESDAY, OCTOBER 9, 1894.

Called to order by the President.

Opened with prayer by Rev. Henry McDonald.

Address of welcome was made by Willis F. Westmoreland, Atlanta.

Response by R. M. Cunningham, Birmingham, Ala.

Telegrams were read from Dr. W. Gill Wylie regretting that, owing to illness of Mrs. Wylie, he could not attend, but that his paper would be sent; from Dr. Jno. A. Wyeth, that he and Dr. A. R. Robinson would arrive this afternoon; from Dr. W. E. B. Davis, that he would get here this noon.

Reports of Secretary and Treasurer were read and referred to Auditing Committee. The reports showed the receipts at the meeting of 1893 to be $240. There was paid out for printing $71.00; to Secretary, $99.01; postage, stationery, etc., $28.84; for hall, etc., $41.15. Total, $240.

Dr. J. C. LeGrand read a paper on "The Responsibility of a Class of Criminals from a Medico-Legal Point of View," in which he advocated castration for capital crimes instead of hanging. The object of this is twofold: first, as a punishment and reformatory measure; second, to prevent the increase of the criminal class. He did not believe that hanging has the deterring effect that castration would have. This treatment cannot be applied except to those convicted of capital crimes.

Dr. R. M. Cunningham said: Crime is a violation of the law—organic, statutory, or common—of the commonwealth in which the criminal lives and to which he is responsible. These laws are the expression of the civilization of the commonwealth.

There is no universal law to define the degree and punishment of crime. There is no universal moral standard. Hence, the responsibility of criminals should be considered in connection with their immediate environment.

External influences alone do not make or prevent criminals. Under precisely the same environment of law—social, ethical, religious, and moral—we have some committing crime and others not. Why so? It is due to some internal, personal agency, that stimulates to the commission of crime, or that restrains the moral inhibition of crime—in either case interrupting the mental, moral, and physical equipoise which in one insures honesty and obedience to law and order, and in the other the violation of same. Whence comes this principle? Partly from race, partly from nationality, and very largely from family.

External influences determine the *yield* of good and evil. Alone they will not make a good man bad, nor *vice versa.*

I think the real cause of crime is found in the criminal himself. When such a principle is discovered, it is the duty of the law to protect society against the propagation of this principle, by emasculation of the criminal, and thereby prevent any posterity.

Dr. J. B. Cowan said: There is no diversity of laws of civilized countries in regard to rape. It is always ranked with murder. The tendency to crime is not alone in the man, but also due to environment. The question takes us into the domain of theology. The rapist is insane. He is irresponsible.

Dr. F. W. McRae thought with Dr. Cowan, that the moral element and environment enters largely in the question. The important point is the prevention of the propagation of this class. Both sexes should be desexualized. It would, however, make this class enemies of society.

Dr. L. P. Brouillette read a paper on, "Treatment of Stricture of the Urethra by Electrolysis," in which he reported a case in which the urethra was impermeable. There was no pain or interference with business. The treatment is limited to stricture due to inflammatory action. He quoted Newman, as to method, differing in that he gave séances on alternate days. He insisted on the permanency of the cure when properly administered.

Dr. J. B. Cowan had had no bad results from the older methods.

All cases had been relieved and most cured. He would be unwilling to throw away the knife in all cases.

Dr. F. W. McRae was opposed to the method, mainly through the report of Dr. Keyes of New York, who states that the results are no better than by rapid dilatation. Had no personal experience with the method. His method was to incise the stricture, and after healing to dilate. The New York Academy of Medicine appointed a committee to investigate the question, and after a year they reported the method a failure.

In closing the discussion, Dr. Brouillette said that the committee did not know anything of electricity and were prejudiced, and this is true of those who oppose this method. He failed only in congenital stricture. This is the best method for treating stricture. The adventitious tissue is absorbed by catalytic action.

AFTERNOON SESSION.

A paper was read by Dr. Frank Trester Smith on "The Induction of Labor to Prevent Blindness." The paper was mainly historical and advocated the induction of labor when blindness was threatened, because this indicated that there was kidney disease of so grave a character as to threaten life of both mother and fetus.

Dr. J. A. Goggans said that he had seen a number of cases with albuminuric retinitis, and that they usually went blind and died.

Dr. J. B. Cowan said that we could not be sure that the albuminuric retinitis was due to the pregnant state. He did not believe in inducing labor promiscuously.

Dr. Smith, in closing the discussion, said that when we had a case of increasing blindness in the pregnant state, we could be quite sure that it was due to the pregnancy; this was more certain if the blindness was increasing rapidly, and especially, if in previous pregnancies there had been attacks of blindness.

A paper by Dr. E. Van Goidtsnoven was read on "Slaughter of the Innocents." (See page 531.)

Dr. R. R. Kime said that we should be careful from a moral standpoint. He did not condemn *in toto* the induction of abortion for the vomiting of pregnancy; still it was getting too common. Instead of a craniotomy, a Cæsarean operation, a symphyseotomy, or a Porro's operation. (In some cases artificial labor is necessary.) In some cases of kidney affections with uremic symptoms, if we wait, the woman will be dead as well as the child.

Dr. R. M. Cunningham could not agree that there were no cases where we should induce artificial labor. The relation between fetus and mother was different from that between child and mother. The fetus was simply a vitalized neoplasm, and should be removed only to preserve life. Craniotomy is an operation that should be obsolete, and is with all scientific men.

Dr. W. E. B. Davis said that those who had performed the most successful operations advocated that in extra-uterine pregnancies early operations should be performed. They urge this to save the mother's life.

Dr. Frank S. Parsons read a paper on "Pneumonia in Children." (See page 537.)

Dr. R. M. Cunningham had treated four or five hundred cases. It was a self-limited disease. He had so found it; about twenty-eight per cent. of his cases had a tendency to die and did die. In many of his cases, heart clots, which he believed to be the cause of death. His method of treatment was by hypodermoclysis. He also used strychnine.

Dr. Parsons, in closing, said that care was necessary in the use of digitalis. Cold was contraindicated in children, as they shuddered from the cold, and increased the action of the heart.

Dr. R. R. Kime read a paper entitled, "Essentials of Obstetric Nursing." Asepsis on the part of physician, patient, and nurse is required. Asepsis may be attained by cleanliness or antiseptics. It is essential that the physician and nurse have clean clothing, hands, and finger nails, and everything coming in contact with patient be rendered aseptic and kept so during the puerperium, even the patient herself. Advised washing and boiling of all cloths, towels, and bedding to be used about the patient. Advised use of antiseptic vulval pad with abdominal binder. Condemned vaginal douche after normal labor. Advised use of catheter by sight after cleansing parts and not by touch, so as to prevent infecting bladder.

Condemned astringents to nipples before labor. Believed infection is more common than most physicians admit, in both city and country practice. That the majority, not all, cases of infection can be prevented by due diligence on the part of the physician and nurse. That many cases of mild infection pass unnoticed by the physician because they are not attended with severe constitutional disturbances, or are classed as malaria, milk fever, taking cold, inflammation of bowels, typhoid fever, etc.

Dr. F. S. Parsons agreed in the main with the author, except that he thought that so much antiseptic material was not necessary. Hot water was the best antiseptic, and all that was needed ordinarily. He used less antiseptics afterwards, but placed the patient in a semi-sitting posture. He advocated immersing the child in warm water, and washing it under water. To prevent excoriation of the naval he wrapped the cord in absorbent cotton. Over this he placed some adhesive plaster. Did not use a binder in any form.

Dr. Kime said that he had not recommended antiseptics except to cleanse the hands of the doctor and the vulva. All cloths should be boiled. The use of the vulval pad and the cotton was largely for the comfort of the patient.

Dr. J. A. Goggans read a paper on "The Surgical Treatment of Empyema." The methods are: aspiration, rib resection, and the open method. The natural tendency is more favorable in children. Empyema is a symptom of various diseases, not a disease itself. Aspiration has many advantages in childhood. He reported thirteen cases operated on mostly by aspiration. He advocated the operation of Porro. The conclusions were as follows:

1. That it is useless to delay the operation of thorocotomy, especially after finding that the pus contains the bacillus of tubercle, or is the result of microbes being introduced through the lymphatics, or when the empyema is developed in cases of general pyemia.

2. That the opening should be made above the ninth rib.

3. That the antiseptic injections should be used only in the above mentioned varieties of empyema.

Dr. J. M. Matthews read a paper on "Some Points in Rectal Surgery." Fistula in ano is one of the most important of diseases of the rectum. It requires more delicate and precise surgery than almost any surgical affection. Irritable ulcer or fissure can be cured by divulsion of the sphincter. At the International Medical Congress, at Washington, he read a paper on "The Anatomy of the Rectum in Relation to Reflexes," in which he endeavored to show that much elucidation could be thrown upon many suspected diseases, by tracing their origin to disease of the rectum. This idea has been run away with by the so-called orificial surgeons, who have done much harm by their method of removing an inch or two of the lower part of the bowel. This practice cannot be too severely condemned.

In ulceration of the rectum sixty per cent. is due to syphilis; simple ulceration is extremely rare. In cancer of the rectum, lumbar colotomy is to be preferred to inguinal. When practicable, the growth should be removed. In the majority of cases, colotomy is unwarrantable.

Dr. W. E. B. Davis said that every gynecological case should have the rectum examined. In cancer of the rectum, unless we can promise that the disease will not return, an operation will do surgery no good.

Dr. F. W. McRae said that the only cases of stricture of the rectum he had seen were due to syphilis. He reported a case where he had performed inguinal colotomy, and had also removed the cancerous growth. This case had been treated for piles. In another case a spasmodic stricture of the urethra was relieved by divulging the sphincter.

Dr. Wm. Perrin Nicolson related a case of ulcer of the rectum in a colored woman, due apparently to dysentery. In a case here some orificial surgeons were going to remove the lower part of the rectum which yielded to iodide treatment.

NIGHT SESSION.

Dr. M. B. Hutchins read a paper on "Mixed Infection," in which he related a case in point.

Dr. A. R. Robinson said that cases of doubtful diagnosis should not be cauterized.

Dr. Hutchins said that many thought the chancroid was merely a pus infection, as no germ had been discovered.

Dr. Dunbar Roy read a paper entitled "Paresis and Paralysis of the External Rectus of the Eye, with Report of Two Cases." In one case the trouble was due to malaria, shown by its yielding to antimalarial treatment. In the second case the paralysis followed a blow on the temple. A cure was effected by gymnastics and electricity. He reviewed the literature of malarial eye diseases, and the paralysis caused by trauma.

Dr. Frank Trester Smith said that he had never known a case of paralysis of the eye muscles to be ascribed to malaria, but the result showed that the conclusion probably was correct.

Dr. G. W. Drake asked if, in the second case, the paralysis was caused by a hemorrhage, where the hemorrhage was located, in the muscle or in the brain.

Dr. Roy thought that there was some effusion in the muscle, and that the center in the brain may have needed some impulse.

Dr. G. W. Drake read a paper entitled "The Code of Medical Ethics." The basic principles upon which the grand old edifice of medicine are builded are "beneficiary and professional liberality," community of knowledge, open medicine, and no therapeutic secrets. He who violates these principles by dispensing secret medicines is a medical heretic or anarchist. He divided all doctors into two classes, regulars and irregulars. He is an irregular who uses any sectarian name or term denoting that his practice is based on any exclusive dogma; he is a regular, whether he prescribes little or large doses, or no doses, whether he employs the principle of "similia" or "contraria," etc., provided he does not assume any ostentatious epithet by which to attract the unwary public, such as eclectic, homeopathic, hydropathic, Indian, hoodoo, etc.

SECOND DAY, WEDNESDAY, OCTOBER 10, 1894.

Called to order by Vice-President J. A. Goggans.

Telegram from Dr. Chas. A. L. Reed announced that he would be unable to attend.

A letter from the President of the Mississippi Valley Medical Society was read, inviting the members to attend their next meeting.

The Auditing Committee reported books of Secretary and Treasurer correct.

Dr. Geo. R. West read a paper on "Uterine Cancer," in which he apologized for introducing a much-talked subject. The cause or origin of cancer is unknown. There is no specific cancer cell. The diagnosis of uterine cancer cannot be made out early except by the corroboration of many vague symptoms, which are usually neglected. The treatment is unsatisfactory, as no cure is known. The best method of procedure is the entire removal of the disease by the knife. The treatment to be effective must be undertaken early, and is, therefore, dependent upon an early diagnosis, and this responsibility usually devolves upon the family physician, into whose hands the patient first falls. It is wrong to declare the inoperable cases doomed, and refuse them treatment. Palliative measures of treatment are suggested. The varieties of operative procedures are mentioned, with preference given to vaginal hysterectomy when the diagnosis is certain. Much time and labor has been expended in research on the subject, and yet the results are so unsatisfactory that it offers a great field for investigation. There is a cure for cancer, and it must be discovered. Who will be the lucky man?

Dr. W. E. B. Davis said that, as a rule, these cases came to the surgeon too late to remove all the disease. The time for the operation is in the early stage. We cannot be sure that the trouble is confined to the cervix, so that total removal is generally indicated. The microscope, in proper hands, is a valuable aid to diagnosis.

Dr. J. M. Matthews said that hysterectomy was not in his line, but cancer was in the line of every surgeon. Cancer is a disease which requires great care in diagnosis. There were cases operated on which should be let alone. He advised that to quiet pain, opium should be used where pain is a factor in the case. He had five cases in which a mistaken diagnosis of carcinoma was made by competent microscopists. He would rather rely on the clinical history.

Dr. John A. Wyeth said that the hope of the profession lay in early diagnosis. This is the secret of success. Operation is simple at that time, and successful. Cancer is a local disease, the result of irritation. When in doubt it is best to operate.

Dr. W. G. Bogart believed the early diagnosis of cancer to be of great importance. In fact, upon the early diagnosis depends our good results. He insisted upon a more thorough study of our cases. Morphia in these cases was to be used sufficiently to overcome pain. We should not neglect to have a microscopical examination made to confirm the general and physical signs we have already had to make us suspect cancer.

Dr. G. W. Drake thought that we should depend on the microscope for the diagnosis. The trouble may be with the microscopist. When in doubt, he would send the case to one who had a large experience with the microscope.

Dr. J. B. Cowan asked if the fault was not with the general practitioner, in that the diagnosis was not made early. He understood that microscopy was a fixed science. The trouble was with the man who handled the instrument.

Dr. A. R. Robinson said that the trouble often was with the specimen, which was not properly prepared, and often included only some curetted material. As a rule, the microscopist, if of sufficient experience, could make the diagnosis. He believed the disease to be infectious.

Dr. G. R. West said that he believed in relieving pain. He wrote the paper because so many cases presented themselves too late for operation.

The President read an invitation to attend the unveiling of the statute of J. Marion Sims. Motion was carried that we have a representative present.

Dr. W. E. B. Davis read a paper on "The Treatment of Stone in the Kidney," in which he pointed out the methods of diagnosis, and dealt with the technique of operating for the relief of the condition produced by stone. He usually makes incision in lumbar region, but if tumor is too large to be removed through this incision, extends it toward the median line. He was working on an instrument by which the urine could be collected from each ureter separately without catheterization of the ureters. He reported a case resembling stone in the kidney, which was due to ectopic gestation. A nephrotomy should be performed rather than a nephrectomy, if there is much healthy tissue.

Dr. John A. Wyeth said the only way to determine the diagnosis, was by an incision, as the author recommended. He sometimes made a T-shaped incision, as it gave more room.

Dr. J. McFadden Gaston reported a case where he had operated for abscess and had found stones impacted in the ureters.

Dr. Davis said that where there was stone in the ureter, it could best be removed by two incisions, one anteriorly to determine location and the other behind for its removal. He thought harm would come from the frequent catheterization of the ureters.

Dr. A. R. Robinson read a paper, "The Importance of Early Treatment in Cutaneous Cancer," illustrating the subject with diagrams.

The earlier the treatment, the less tissue to be removed and the greater the chances of cure. The superficial discoid form may disappear, may grow very slowly, or may grow rapidly, and all should be operated on. The papillary may begin as a wart or mole, or may be secondary to the superficial discoid. Early treatment is more strongly called for. The deep-seated or nodular should be diagnosed early and removed. It first appears as a small, hard nodule under the skin, gradually increasing in size. The deep tissue should be removed.

Dr. M. B. Hutchins advocated the removal of every suspicious growth, for we could not tell which would be dangerous.

Dr. John A. Wyeth said that he would not rely on the knife alone, but used caustics. In the nodular form he would not begin with the paste. He related a case removed by incision three times, and recovery took place after the use of Marsden's paste. In another case, cure took place after an attack of erysipelas. He believed the disease due to a germ, and that a cure would be found.

Dr. J. McFadden Gaston related a recent case of large epithelioma on the face removed by the electro-cautery.

Dr. J. B. Cowan said that the idea of non-interference pervaded not only the laity but also the profession.

Dr. P. L. Brouillette related a case of epithelioma of twenty-five years' growth which he removed. He emphasized the necessity of early treatment.

Dr. A. R. Robinson did not recognize a recurrence in these cases. It was generally a reappearance.

On motion, an invitation to attend an operation by Dr. Wyeth, at 9 A. M. to-morrow at the Southern Medical College, was accepted—the Society to meet immediately after.

Dr. John A. Wyeth read a paper on "A Report of Some Rare Surgical Lesions Connected with the Liver." (See page 523.)

Dr. W. E. B. Davis said that cases of obstruction of biliary ducts should be operated on rapidly—can't stand long operation. The best method is incision, the insertion of a tube and packing with gauze. Do not take time to sew up duct.

Dr. J. McF. Gaston indorsed external drainage, as advocated by Dr. Davis.

On motion, President's address was made a special order for 4 P. M.

AFTERNOON SESSION.

Vice-President Howell presiding.

A paper by Dr. C. H. Holland on "Treatment of Small Pox," was read by title.

Dr. Ellis Drewry read a paper on "Burns, and Treatment Thereof," in which he advocated antiseptics. The indications are to allay irritation, reduce pain, prevent sepsis.

Dr. W. G. Bogart said that no class of injuries are more serious or difficult to obtain good results from than burns.

The steps indicated in the treatment of burns are:

1. Relief of pain.
2. Overcome shock.
3. Dressing injury.

First indication is met with hypodermic injection of morphia.

2. Strychnia, or alcoholic stimulants.

3. White lead, iodoform with vaseline, etc.

Report of two burns, the result of grasping an electric wire.

Dr. F. S. Parsons said that ether was a good remedy to overcome shock. In Philadelphia this is the practice in the hospitals. Recently bovinine has been used in burns of the second degree. The result is that the granulations are made healthy.

Later grafts from the superficial part of the skin were placed over the burn to promote healing. Hard callosities were good for this purpose.

Dr. Frank Trester Smith asked how corns would do.

Dr. Frank S. Caldwell said that he could answer that, in the railroad hospital he had used corns. In small burns they used camphophenique, but in large burns carbolic acid and olive oil. In one case where a recent graduate had placed cotton next the burn, he was two years in getting rid of the cotton.

Dr. Drewry related a case in which it took him six months to get rid of a cotton dressing.

Dr. J. B. S. Holmes delivered the President's address, "Some Causes Leading to Invalidism in Women." He protested against the amount of work required of girls at schools. The fault lies with the parents, who want them to graduate at an early age. The system sends cases to the doctor. He suggested that the girls be treated as boys until the time of menstruation. The mother should inform the daughter of the expected change. She should be taught to keep quiet during the flow three or four years.

Among the causes he mentioned, the study of music, high heels, exposure at time of menstruation, infection at childbirth from failure to repair a tear, or from applications by the surgeon, gonorrhea.

On motion, the thanks of the Society were extended to the retiring President for his excellent address.

Dr. Author G. Hobbs, read a paper entitled "Adenoids and their Treatment with a New Forceps." The means used in treatment are the ring knife of Meyer, the finger which is dangerous on account of the danger of getting material in larynx, Volkman's curette, the wire loop gouge, and forceps, which is the most rational method. He presented an original forceps for the removal of these growths.

Dr. J. A. Goggans presented a specimen of extra-uterine pregnancy. The case, when first seen, was diagnosed as extra-uterine pregnancy with ruptured sac, but operation was refused until a month later. Abdomen was full of blood. Sac firmly adherent in pelvis, hemorrhage controlled by gauze packing. Death took place thirty-six hours later.

NIGHT SESSION.

At night the Society attended a reception given by the medicai profession of Atlanta, at the Capital City Club.

THIRD DAY, THURSDAY, OCTOBER 11, 1894.

The Society witnessed an operation by Dr. John A. Wyeth, of New York, for the removal of a large cyst of the thigh, at the Southern Medical College, after which it was called to order at the Kimball House. The Council reported the following nominations for officers and they were duly elected :

President—R. M. Cunningham, Birmingham, Ala.

First Vice-President—J. C. LeGrand, Alabama.

Second Vice-President—Floyd W. McRae, Georgia.

Third Vice-President—G. W. Drake, Tennessee.

Secretary—Frank Trester Smith, Chattanooga.

Treasurer—George R. West, Chattanooga.

The President-elect, also Vice-Presidents LeGrand and McRae were introduced and addressed the Society.

The following papers were read by title :

"Reflex Neurosis in the Male," Andrew Boyd, Scottsboro, Ala.

"Excision of Malignant Tumors of the Breast," W. F. Westmoreland, Atlanta.

Dr. Valentine H. Taliaferro read the paper on "Transfusion of Blood and Infusion of Salines." He advised the operation for hemorrhage and also for shock. He used a saline solution heated to 110° F. He had operated five times and had seen a number of cases and had never seen any harm. He reported cases where the measure was successfully used where death seemed imminent.

Dr. Wm. Perrin Nicolson said the operation could be performed safely. In McBurney's hospital, New York, this measure is carried on simultaneously with the operations. He knew of cases where the temporary effect was good, but death took place later.

Dr. Frank Trester Smith called attention to a paper read before this Society by R. M. Cunningham and published in the *Virginia Medical Monthly*, 1893, in which the method of hypodermoclysis was described.

Dr. Taliaferro, in closing, said that injections into the cellular tissue were to be condemned on account of danger of abscess, etc. The method by high injection into the rectum was not as good as injection directly into the veins.

Dr. Wm. Perrin Nicolson read a paper entitled "Combination of Carbolic Acid and Camphor as an Antiseptic and Local Anesthetic." He recommended it for all wounds, for pruritus, and for parastic diseases. It causes no reaction and there is no absorption. Catgut was kept soft and aseptic a year in the mixture.

Dr. R. R. Kime said that he had used the following preparations of camphor and carbolic acid: Camphorated phenol is made of carbolic acid, to one or two parts gum camphor. Camphorated iodo-phenol is made by adding tincture iodine to camphorated phenol in the proportion desired, to which glycerine may be added. Camphorated salicylo-phenol is made by adding salicylic acid to camphorated phenol in proportion desired. Boric acid may also be combined with phenol in small proportions.

Has used camphorated phenol for years, considers it a positive germ destroyer. It checks putrefaction at once, as well as pus formation. It also checks septic invasion when it can be brought in contact with the germs and foci of infection. Its burning and irritation can be prevented by increasing the proportion of gum camphor.

It is the best remedy we have to apply to uterine cavity after curetting or removal of putrid-septic or purulent material, swabbing out with cotton, and on gauze packing. It instantly checks the putrefaction, etc. It is the best remedy to check pus formation in all suppurating wounds, and is excellent in burns, erysipelas, carbuncle, and dressing wounds.

Prefers the name camphorated phenol in contradistinction to the proprietary preparation known by the name Campho-phenique. Had published an article about one year ago setting forth the combinations and uses of gum camphor and carbolic acid.

Dr. R. P. Johnson thought that by mixing carbolic acid, camphor, and vaseline at the same time cauterization would result.

Dr. Nicolson related some cases where union by first intention took place in extensive wounds.

Dr. R. P. Johnson read a paper on "Headaches; Their Etiology and Treatment."

### AFTERNOON SESSION.

Resolutions were adopted indorsing the efforts now being made for the prevention of blindness from ophthalmia neonatorum by legislative enactment.

Resolutions of thanks were adopted to the Railroads, the Kimball House, Dr. Westmoreland, the President, the Secretary, the profession of Atlanta, and the daily papers.

A paper by Dr. E. A. Cobleigh, "Pernicious (or Inveterate) Vomiting of Pregnancy. A Plea for the Mother Based on Cases in Actual Practice," was read by title.

The officers were then installed, and after prayer by Dr. J. B. Cowan, the Society adjourned to meet in Chattanooga the second Tuesday in October, 1895.

---

### PERSONAL NOTES.

Dr. J. B. Cowan, of Tullahoma, Tenn., is one of the members who will never grow old. His genial manners and youthful spirits will long be remembered by his many friends.

Dr. Parsons, of Philadelphia, was in attendance. This was his first visit to the Society, and the members were glad to make his acquaintance.

Dr. A. E. Robinson, the eminent skin specialist of New York, was one of the visiting members. Dr. Robinson is one of the faculty of the Polyclinic, and was known to many of the members.

Dr. R. M. Cunningham, who was elected President by the Association, deserved the honor he received. He was the life of the Association, and his election to the position of President showed how he was appreciated by his fellow-members.

Dr. J. B. S. Holmes, the retiring President, is one of the new citizens of Atlanta, of whom we are justly proud. Dr. Holmes's magnificent new sanitarium will soon be completed and will no doubt be filled from the opening.

Dr. John A. Wyeth, the President of the New York Polyclinic, and New York's foremost surgeon, was at the meeting, and operated before the members in the amphitheater of the Southern Medical College. Many of the members were formerly attendants at the

Polyclinic in New York, and vied with each other in making the doctor's visit a pleasant one.

Dr. C. B. Crawford, of Augusta, was the only representative from that city present. The many friends of the doctor were glad to see him in attendance.

Dr. G. W. Drake, of Chattanooga, was present, and added much to the success of the meeting.

Dr. T. Ellis Drewery, of Griffin, was present, and his paper on "Burns" attracted much attention.

Dr. Floyd Wilcox McRae, of Atlanta, was in attendance the whole of the session, and added much to the entertainment of the visiting members.

Dr. J. M. Matthews, the great specialist of Louisville, whose fame as an author and operator is world-wide, was on hand. Dr. Matthews is one of the bright lights of the profession, and is always welcome here.

Many thanks are due to the efficient Secretary, Dr. Frank Trester Smith, for his untiring efforts in adding to the success of the meeting. The members showed their appreciation of his work by re-electing him Secretary.

NOTES OF THE SOCIETY.

The exhibit of Messrs. Parke, Davis & Co. was a very fine one. This enterprising firm is always at the front, and this fact, combined with the elegance and reliability of the products of their laboratory, have been the factors in building up their enormous business in the South. Their exhibit was in charge of Mr. D. R. Stauffacher, who is well and favorably known by the doctors of the South. Mr. Hamilton was on hand as one of their representatives, and they did credit to the house they represent.

Messrs. Wm. R. Warner & Co., represented by Mr. Chears, who is one of the most popular of salesmen, had an exhibit eminently worthy of this old and established house. Their goods were well shown, and every one knows that their preparations are strictly first-class. Mr. Chears has been with Messrs. Warner & Co. for years, and it was he who furnished the bromo soda, without which the morning sessions would have been a complete failure.

Messrs. Sharpe & Dohme had a display of their products. This firm has a large Southern patronage. Their goods are strictly first-class and need no praise from us. Messrs. Sprague, Pryor, and Curtis represented them, and three better men could not be found. Mr. Sprague is soon to be married, and each of his friends among the members of the Tri-State Medical Society wishes him a long life and much happiness.

Messrs. Frederick Stearnes & Co. had one of the most elaborate displays that was shown. Their preparation of wine of cod liver oil and peptonate of iron is one that is gaining ground very rapidly. This firm's trade in the South is increasing daily. Their representative, Mr. Rice, is a good talker, and loses no opportunity to push the interests of his house.

John B. Daniel, of Atlanta, was the one firm that made an exhibit of instruments. Mr. Daniel, by his pluck, energy, and good business management, has built up one of the largest houses of his kind in the South. His trade extends all over the South, West, and part of the Northern and Eastern States.

The New York Pharmaceutical Company was represented by Dr. Pollock. Their old and well known Hayden's Viburnum Compound is known to all, and wherever introduced it at once becomes a fixture.

Messrs. Henry K. Wampole & Co. had a fine exhibit under the care of Mr. Langley and Mr. Ligon. This firm exhibited a fine line of their preparations.

Messrs. W. S. Merrill & Co. was represented by Mr. Justice. Their preparations were well shown and elegantly prepared. This firm makes a full line of fine chemicals and drugs.

The Maltine Manufacturing Co., with their usual push and energy, were represented by Dr. Fite. Their new preparation of "Maltine with Coca Wine" is one of their happiest efforts, and is destined to be one of the best known of all of their preparations. It is one of the latest of the elegant pharmaceutical preparations, and its sale is already enormous. Their other standard preparations of maltine and combinations are well known to all.

The Malted Milk Company was represented by Mr. Bryson. The enormous sale of this preparation, and the way that the business of this firm is increasing in the South, is phenomenal. The doctors of the South are not slow to catch on to a good thing, and the fact that this preparation is one of the best is beginning to dawn upon them. Hence the business this liberal and enterprising firm is establishing in this section.

The old firm of Doliber, Goodale & Co., of Boston, with the "old reliable" Mellin's Food, was on hand. The older this preparation grows the better it seems to get, and when once used, it is always used. This firm is one of the most liberal in the United States, and their product is sold all over the world. They have a large and increasing patronage in the South.

